# From darkness to light: Case report on afamelanotide-treatment in a 9-year-old child with erythropoietic protoporphyria

**DOI:** 10.1016/j.jdcr.2025.11.022

**Published:** 2025-11-24

**Authors:** Anna-Elisabeth Minder, Jasmin Barman-Aksözen

**Affiliations:** a Division of Endocrinology, Diabetes and Porphyria, Stadtspital Zürich, Triemli, Zürich, Switzerland; b Swiss Reference Centre for Porphyrias, Stadtspital Zürich, Triemli, Zürich, Switzerland; c Institute of Laboratory Medicine, Stadtspital Zürich, Triemli, Zürich, Switzerland; d Division of Metabolism and Children’s Research Center, University Children’s Hospital, Zurich, Switzerland; e University Research Priority Program “ITINERARE – Innovative Therapies in Rare Diseases”, University of Zurich, Zurich, Switzerland; f International Porphyria Patient Network (IPPN), Zürich, Switzerland

**Keywords:** afamelanotide, erythropoietic protoporphyria, orphan drug, pediatrics, phototoxicity

## Introduction

We present the case of an effective treatment with afamelanotide in a severely affected 9-year old girl with erythropoietic protoporphyria (EPP).

EPP is an ultra-rare genetic disorder that leads to excess production and accumulation of the heme precursor protoporphyrin IX (PPIX) during erythropoiesis.^1^ In EPP, when the skin is exposed to visible light, PPIX is photoactivated causing phototoxic reactions due to vascular damage by oxygen radicals. These can be very painful (median maximum pain score in the Swiss cohort on visual analog scale 10/10),^2^ develop within a few minutes of light exposure,^3^ may last for several days,^1^^,^^2^ and significantly impair quality of life (QoL).^1^^,^^2^^,^^4^^,^^5^

## Case report

A girl with no family history of photosensitivity or any porphyria suffered from painful phototoxic reactions since early childhood. At the age of 5, she was diagnosed with EPP due to elevated PPIX-levels in the erythrocytes. Until recently, the 9-year-old patient had coped with her light intolerance using light-protective clothing (ie, hat, gloves, long sleeves and long trousers, socks) and further protective measures as needed (ie, umbrella or shades), which provided partial protection from phototoxic reactions. However, after a severe pneumonia in winter, she experienced dramatic worsening of her EPP symptoms the following spring. Despite the usual protective measures, the girl developed a severe phototoxic reaction that did not subside for several weeks. The pain was severe enough to disrupt both her sleep and school attendance. At first, she was able to attend school on an hourly basis only, and later she was unable to attend school at all for 6 weeks, necessitating homeschooling. Her painful reactions were also exacerbated by light passing through the windows, from artificial light and even by the light of a computer screen. Her symptoms required the whole family to live in darkness for several weeks to avoid exacerbating her phototoxicity.

After thorough discussion with the child and her parents, we decided to perform an off-label treatment with a 16 mg implant of afamelanotide, which is approved for the treatment of EPP in adults.

The patient tolerated the medication well and other than mild nausea for a couple of days after the treatment no side effects were noted.

Three days after the treatment, the family gradually started to open their shutters and allowed more light in their home. On the fourth day, the girl attended school for 4 hours for the first time in almost 10 weeks. Since then, she has returned to normal school attendance, even joining her classmates for school trips. Her EPP-QoL score, a validated QoL questionnaire for EPP patients ranging from 0 (lowest possible QoL) to 100 (highest possible QoL),^6^ improved from 0 before the treatment to 55.6 after only 5 weeks.

## Discussion

An increase in light intolerance in EPP can occur after iron substitution, which this patient did not receive. Also, it is often due to a complicating hepatopathy as the excess PPIX is primarily cleared by the liver via the bile with an inherent risk of liver damage (appr. 2% to 4%).^7^ The laboratory tests and the ultrasound in our patient showed no sign of hepatopathy. Moreover, PPIX levels were unchanged compared to the previous years (Table I). Therefore, the reason for the dramatic worsening of the symptoms in our patient remains unclear. However, in the Swiss cohort we have observed a striking increase of photosensitivity in 2 other (adult) patients with EPP after an infection treated with antibiotics. Hence, a causal relationship cannot be excluded, although the potential mechanisms of this are unknown.

**Table I:** Laboratory test results before and 4 weeks after treatment

	Unit	Value 04/2024 (Reference value)	Value before treatment 2025 (reference value)	Value after treatment 2025 (reference value)
PPIX	μmol/l	**8.5** (<0.2)	**8.4** (<0.2)	**8.1** (<0.2)
AST	U/l	34 (<35)	28 (10-47)	31 (17-50)
ALT	U/l	23 (<35)	26 (10-39)	23 (7-44)
Bilirubin	mg/dl		0.3 (0-1.0)	0.2 (0.3-1.2)
Ferritin	μg/l	35 (13-68)		23.3 (22-158)
Hb	g/dl	12.1 (11.2-14.8)	12.8 (9.2-15.5)	12.8 (11.2-14.6)

In bold: pathological values.

*ALT,* Alanine aminotransferase; *AST*, aspartate transaminase; *Hb*, hemoglobin; *PPIX*, free erythrocyte protoporphyrin.

Afamelanotide is approved for the prevention of phototoxicity in adult patients with EPP. It is a synthetic analog of alpha-melanocyte stimulating hormone and a melanocyte receptor 1 agonist. It binds and activates melanocyte receptor 1 on epidermal melanocytes, leading to increased eumelanin production that prevents PPIX photoactivation, along with antioxidative and anti-inflammatory effects. It increases the time patients can spend in direct sunlight without symptoms and reduces the number and severity of phototoxic reactions.^8^ It is available as a 16 mg-controlled release implant. The treatment is generally well tolerated, common side effects include nausea and headaches, also flushing, dizziness or fatigue can occur.

The lack of approved medical treatment options in children with EPP reflects that the burden of this ultra-rare disease often is under-recognized and, therefore, it is extremely difficult to address the medical needs of these patients.

In conclusion, our young patient showed positive responses to the treatment with afamelanotide, allowing her participation in social life, and no significant or unexpected side effects were noted. Hence, we suggest that approved therapies for adults should be evaluated for the treatment of children with EPP.

## Conflicts of interest

Dr Minder received unrestricted research grant 2020 and 2021 by Clinuvel Pharmaceuticals, financial support for a porphyria nurse by Alnylam pharmaceuticals 2022 and 2023, both paid to Stiftung für wissenschaftliche Forschung, Stadtspital Zürich, Schweiz; Dr Barman-Aksözen has no conflicts of interest to declare.
